# Supplementary material for: Combining KPNA2 and FOXM1 Expression as Prognostic Markers and Therapeutic Targets in Hormone Receptor-Positive, HER2-Negative Breast Cancer
Source: Cancers (Basel). 2025 Feb 17;17(4):671. doi: 10.3390/cancers17040671 (PMC11853725; doi:10.3390/cancers17040671)
Supplement: Supplementary file 1 [file cancers-17-00671-s001.zip › cancers-3448404-supplementary.pdf]

Supplementary materials:

a

Tumor Grade (MMH)			
P<0.0001; n=172	I	II	III
KPNA2 low	23	62	3
KPNA2 high	4	57	23

Tumor Grade (GSE7390, Probe: 201088_at)			
P<0.001; n=196	I	II	III
KPNA2 low	23	59	16
KPNA2 high	7	24	67

Tumor Grade (GSE7390, Probe: 211762_s_at)			
P<0.001; n=196	I	II	III
KPNA2 low	23	60	15
KPNA2 high	7	23	68

b

AJCC Tumor Stage (MMH)			
P=0.0107; n=184	I	II	III/IV
KPNA2 low	65	19	8
KPNA2 high	45	31	16

AJCC Tumor Stage (TCGA BRCA)			
P=0.007; n=769	I	II	III/IV
KPNA2 low	83	212	89
KPNA2 high	50	234	101

c

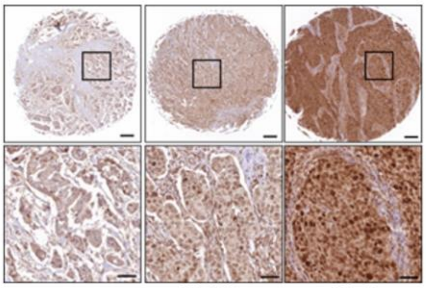

Tissue microarray				
P=0.049; n=54		Tumor Grade		
		I	II	III
KPNA2 Score	Low	0	7	1
	Middle	6	33	4
	High	0	1	2

Figure S1. **Significant association of KPNA2 transcript and protein levels with tumor grades and AJCC stages.** (**a**) Contingency tables summarize the distribution of patients among grade I-III tumors in the MMH and GSE7390 datasets when patients were grouped into KPNA2_high and KPNA2_low subgroups based on cohort median of KPNA2 expression. (**b**) Contingency tables summarize the distribution of patients among AJCC stage I-IV tumors in the MMH and TCGA_BRCA datasets when patients were grouped into KPNA2_high and KPNA2_low subgroups based on cohort median of KPNA2 expression. (**c**) Representative images of immunohistochemistry (IHC) staining of KPNA2 using the GTX106323 antibody (Genetex, San Antonio, TX, USA) on commercial tissue microarray BR803c (US Biomax, Rockville, MD, USA). The KPNA2-specific staining was interpreted and scored by pathologists as low, middle, or high group based on statistical requirements. The *P* values of Chi-square test for trend as well as cohort size (n) were indicated.

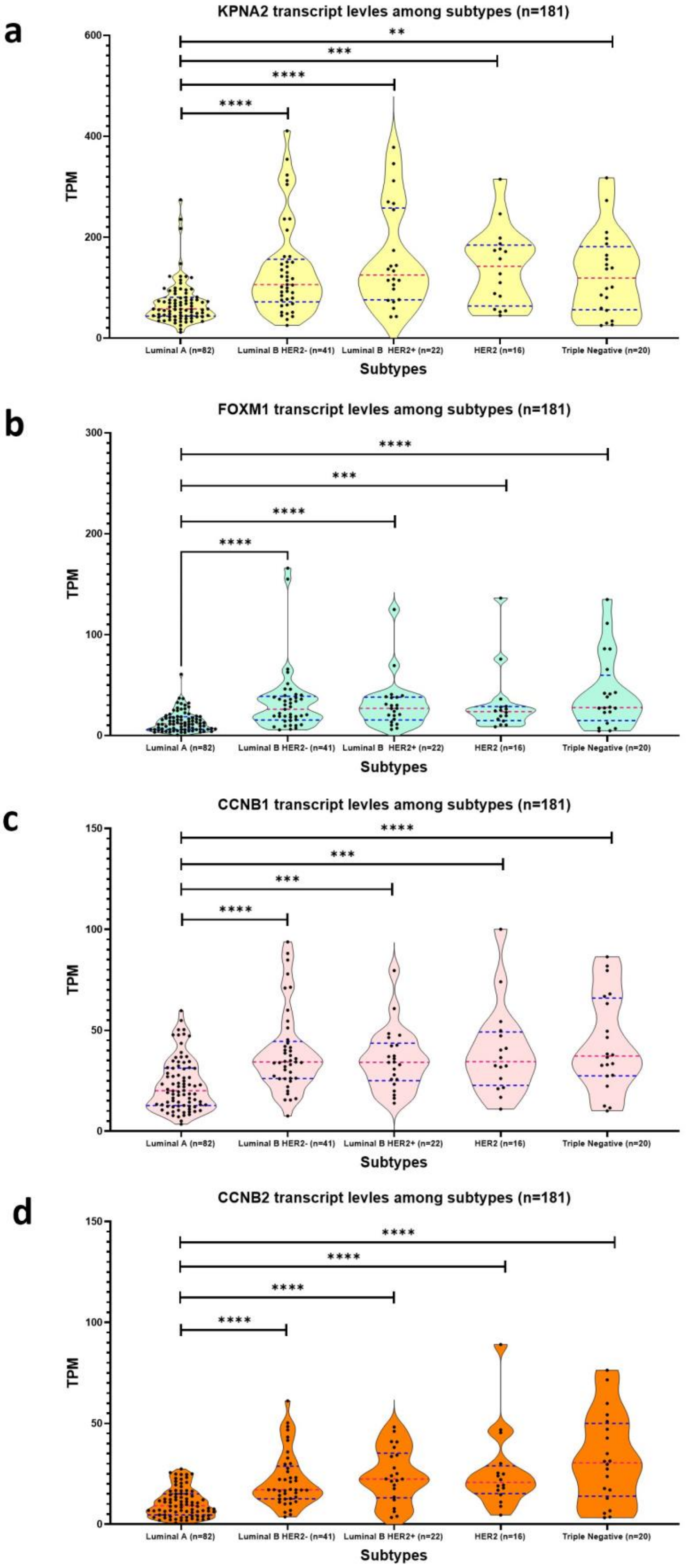

Figure S2. Distribution of the four prognostic gene transcript levels across the intrinsic subtypes of the 184 BRCA patients in MMH dataset. Significant differences (P < 0.05, Mann-Whitney test) between pair-wise subtype comparisons are indicated by a (****, P ≦ 0.0001), a (***, P ≦ 0.001) or a (**, P ≦ 0.01). TPM, transcripts per million.

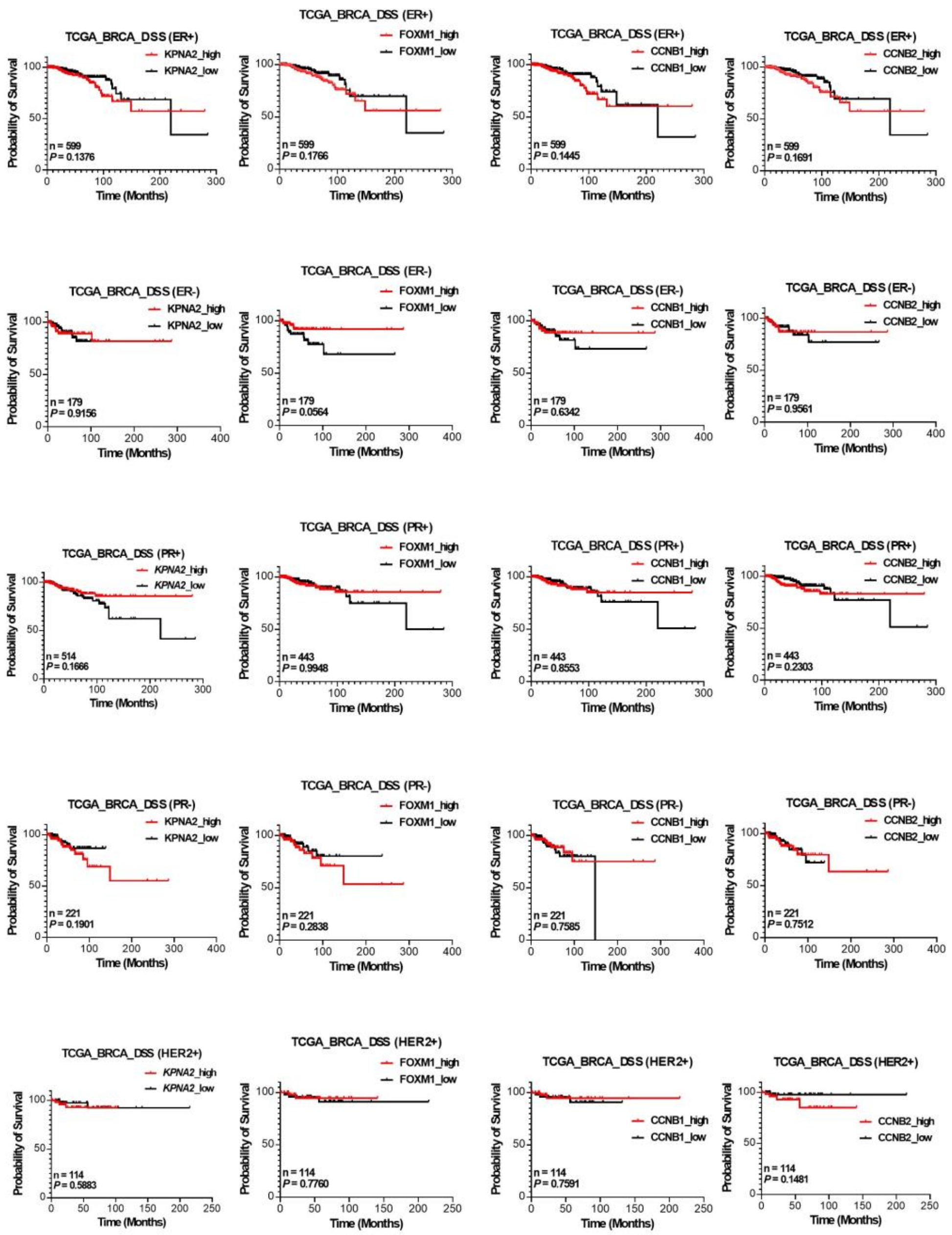

Figure S3. All KPNA2, FOXM1, CCNB1, and CCNB2 were not prognostic of DSS across subgroups of different receptor status, except for subgroup HER2-Negative in the TCGA dataset. The expression levels of KPNA2, FOXM1, CCNB1 or CCNB2 were not prognostic as indicated by the log-rank P values in the Kaplan-Meier survival analysis of disease-specific survival (DSS) in all subgroups of positive or negative status for ER, PR and HER2. DSS: Disease-specific survival; ER: estrogen receptor; PR: progesterone receptor; HER2: human epidermal growth factor receptor 2.

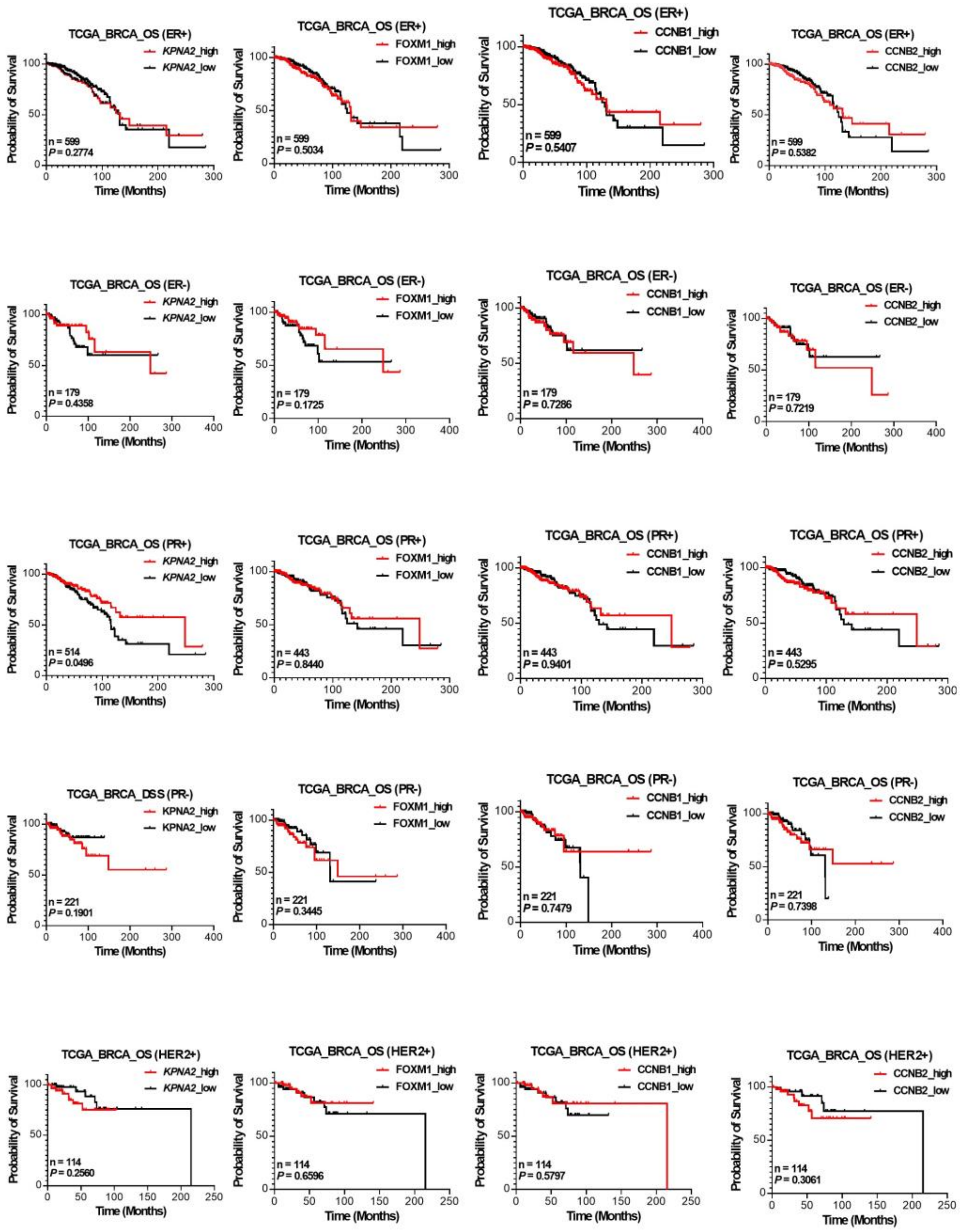

Figure S4. All KPNA2, FOXM1, CCNB1, and CCNB2 were not prognostic of OS across subgroups of different receptor status, except for subgroup HER2-Negative in the TCGA dataset. The expression levels of KPNA2, FOXM1, CCNB1 or CCNB2 were not prognostic as indicated by the log-rank P values in the Kaplan-Meier survival analysis of OS in all subgroups of positive or negative status for ER, PR and HER2. OS: overall survival; ER: estrogen receptor; PR: progesterone receptor; HER2: human epidermal growth factor receptor 2.

Table S1. Gene expression profiling data used in this study. The source data and clinicopathological parameters were downloaded from the UCSC Xena and GEO database.

Cohort	Source	References
TCGA BRCA	UCSC Xena	https://xenabrowser.net/datapages/?cohort=TCGA%20Breast%20Cancer%20(BRCA)&removeHub=https%3A%2F%2Fxena.treehouse.gi.ucsc.edu%3A443
GSE7390	GEO database	https://www.ncbi.nlm.nih.gov/geo/query/acc.cgi?acc=gse7390

Table S2. Patient characteristics of the TCGA_BRCA cohort. Clinicopathological data distribution and association with KPNA2/FOXM1/CCNB1/CCNB2 transcript levels of the TCGA cohort of women with breast cancer whose primary tumors were examined by RNAseq for transcriptome profiling.

Clinical data distribution and association with KPNA2/FOXM1/CCNB1/CCNB2 transcript levels of the 1096 TCGA cohort of women with breast cancer whose primary tumors were examined by RNAseq for transcriptome profiling.

	**Entire cohort (n=1096)**	***P*-values low* expression vs high* expression**			
		**KPNA2**	**FOXM1**	**CCNB1**	**CCNB2**
**Age at diagnosis**					
median (min-max)	58 (26–90)	*P = 0.0058*	*P* = 0.0705	*P* = 0.3628	*P* = 0.0664
**Receptor status**					
ER-positive	601 (55%)	*P < 0.0001*	*P < 0.0001*	*P < 0.0001*	*P < 0.0001*
PR-positive	443 (40%)	*P = 0.8694*	*P = 0.3251*	*P = 0.9347*	*P = 0.2503*
HER2-positive	114 (10%)	*P < 0.0001*	*P = 0.0044*	*P < 0.0001*	*P < 0.0001*
**Intrinsic subtype**		*P = 0.6189*	*P = 0.0059*	*P = 0.4728*	*P = 0.3508*
Luminal A	209 (19%)				
Luminal B	112 (10%)				
HER2-enriched	56 (5%)				
Basal-like	89 (8%)				
**Pathology type**		*P = 0.4066*	*P = 0.4618*	*P = 0.0549*	*P = 0.5268*
Ductal	783 (71%)				
Lobular	198 (18%)				
Mixed	35 (3%)				
Mucinous	16 (1%)				
Others	59 (5%)				
**TNM stage, pT**		*P = 0.0440*	*P = 0.0070*	*P = 0.0011*	*P = 0.0041*

T1	268 (24%)				
T2	646 (59%)				
T3 and above	176 (16%)				
**TNM stage, pN**		*P = 0.3632*	*P = 0.0635*	*P = 0.0941*	*P = 0.1721*
N0	509 (46%)				
N1	376 (34%)				
N2 and above	186 (17%)				
**TNM stage, pM**		*P = 0.6742*	*P = 0.5359*	*P = 0.2054*	*P = 0.0891*
M0	907 (83%)				
M1	23 (2%)				
**Stage by AJCC** ver. 8**		*P = 0.0098*	*P = 0.0003*	*P = 0.0025*	*P = 0.0011*
Stage I	133(12%)				
Stage II	446 (41%)				
Stage III/IV	190 (17%)				

***Low or high KPNA2/FOXM1/CCNB1/CCNB2: KPNA2/FOXM1/CCNB1/CCNB2 transcript levels lower or higher than the cohort median of respective transcript.**

****AJCC: American Joint Commission of Cancer.**
